# Dynamic regulation of phenylpropanoid pathway metabolites in modulating sorghum defense against fall armyworm

**DOI:** 10.3389/fpls.2022.1019266

**Published:** 2022-11-25

**Authors:** Sajjan Grover, Sanket Shinde, Heena Puri, Nathan Palmer, Gautam Sarath, Scott E. Sattler, Joe Louis

**Affiliations:** ^1^ Department of Entomology, University of Nebraska-Lincoln, Lincoln, NE, United States; ^2^ Wheat, Sorghum, and Forage Research Unit, U.S. Department of Agriculture-Agricultural Research Service, Lincoln, NE, United States; ^3^ Department of Biochemistry, University of Nebraska-Lincoln, Lincoln, NE, United States

**Keywords:** sorghum, fall armyworm, plant defense, flavonoids, regurgitant

## Abstract

Plants undergo dynamic metabolic changes at the cellular level upon insect infestation to better defend themselves. Phenylpropanoids, a hub of secondary plant metabolites, encompass a wide range of compounds that can contribute to insect resistance. Here, the role of sorghum (*Sorghum bicolor*) phenylpropanoids in providing defense against the chewing herbivore, fall armyworm (FAW), *Spodoptera frugiperda*, was explored. We screened a panel of nested association mapping (NAM) founder lines against FAW and identified SC1345 and Ajabsido as most resistant and susceptible lines to FAW, respectively, compared to reference parent, RTx430. Gene expression and metabolomic studies suggested that FAW feeding suppressed the expression level of genes involved in monolignol biosynthetic pathway and their associated phenolic intermediates at 10 days post infestation. Further, SC1345 genotype displayed elevated levels of flavonoid compounds after FAW feeding for 10 days, suggesting a diversion of precursors from lignin biosynthesis to the flavonoid pathway. Additionally, bioassays with sorghum lines having altered levels of flavonoids provided genetic evidence that flavonoids are crucial in providing resistance against FAW. Finally, the application of FAW regurgitant elevated the expression of genes associated with the flavonoid pathway in the FAW-resistant SC1345 genotype. Overall, our study indicates that a dynamic regulation of the phenylpropanoid pathway in sorghum plants imparts resistance against FAW.

## Introduction

Sorghum [*Sorghum bicolor* (L.) Moench] is one of the top five cereal crops cultivated in the world and the third most cultivated grain in the United States. Sorghum is well known for its versatile role as cereal crop, livestock feed, and bioenergy source. However, more than 150 species of insects have been reported to cause economic losses in sorghum (Nwanze and Youm, 1995). The fall armyworm (FAW), *Spodoptera frugiperda* (J. E. Smith) (Lepidoptera: Noctuidae), is one of the most important chewing pests of sorghum in North and South America. Due to its polyphagous nature, voracious feeding habits, and wide host range of ~353 plant species, FAW has expanded its geographic range to other parts of the world (Montezano et al., 2018). The FAW larval stage feeds on the leaves, whorls and makes large and irregular holes in the leaves and sometimes leaving only the midrib behind (Chamberlin and All, 1991). The application of synthetic insecticides to control insect pests under field conditions is solely an immediate solution but is not environmentally safe. The imprudent and continuous usage of insecticides can also lead to the development of insecticide resistance in a short time and reduce the diversity of natural enemies. Host plant resistance, which focuses on plant defense mechanisms in response to insect attack, is considered as a promising and ecologically sound tool among pest management strategies (Panda and Khush, 1995).

Plants can induce a suite of defense pathways against various insect pests and these defenses are frequently conserved among plant species against different insects. The phenylpropanoid pathway – a hub of secondary metabolites, encompasses an array of molecules such as monolignols, flavonoids, coumarins, phenolic glycosides, phenolamides, benzoic acids, stilbenes, with a wide range of important functions, especially in plant-environment interactions (Fraser and Chapple, 2011). The role of phenylpropanoids in plant defenses ranges from physical to chemical barriers against insects/pathogens, and phenylpropanoids can act as signal molecules involved in local or systemic signaling for activating defense mechanisms (Alon et al., 2013). Phenylpropanoids can act as antifeedants, oviposition deterrents, and toxic poisons; although some of these compounds may also act as stimulants to attract insects to feed and reproduce (Dixon et al., 2002; Bernards and Båstrup-Spohr, 2008).

The plant cell wall, a first line of contact between the internal and the external environment, often contains lignin which is formed by formed the oxidative polymerization of monolignols, primarily p-coumaryl, coniferyl, and sinapyl alcohols. Several reports showed a positive correlation between lignin deposition in the cell wall and enhanced resistance to insects, pathogens, or mechanical wounding (Ride, 1980; Hématy et al., 2009; Moura et al., 2010; Santiago et al., 2013). The phenylpropanoid pathway that synthesizes monolignols also generates a wide range of phenolic compounds that serve as chemical precursors for resistance to insects/pests and various other plant functions. Deamination of phenylalanine by phenylalanine ammonia lyase (PAL) generates *p*-coumaric acid, the first dedicated precursor for lignin and other related phenolic biosynthesis (Scully et al., 2016). Therefore, the modulation of genes involved in this pathway impacts lignin and synthesis of various phenolics (Baxter and Stewart, 2013). Flavonoids, a wide group of secondary metabolites, which include six major groups: chalcones, flavones, flavonols, flavandiols, anthocyanins, and the condensed tannins are derived from phenylalanine and malonyl-coenzyme A (Panche et al., 2016). Recently, flavonoids have also been found effective against aphids and chewing type insects (Morkunas et al., 2016; Kariyat et al., 2019; Zogli et al., 2020; Stec et al., 2021; Zhang et al., 2022). Moreover, flavonoids act as non-enzymatic antioxidants to suppress ROS generation and protects plants against ROS auto-toxicity (Bernards and Båstrup-Spohr, 2008; Baskar et al., 2018).

Plants have developed a sophisticated system to recognize insect cues and activate the defense responses against them (Fürstenberg-Hägg et al., 2013; Abdul Malik et al., 2020). Chewing insects cause extensive damage to plants, and they also release regurgitant and saliva while feeding (Turlings et al., 1993; Alborn et al., 1997; Chuang et al., 2013), which shape the plant response to herbivores (Chuang et al., 2013; Ray et al., 2015; Acevedo et al., 2019; Grover et al., 2022a). Application of caterpillar regurgitant can induce plant defense responses (Chuang et al., 2013; Acevedo et al., 2019). Regurgitant arises from the digestive system of the insect and changes in plant defense responses have been mainly attributed to fatty acid-amino acid conjugates present in the regurgitant (Pohnert et al., 1999; Yoshinaga et al., 2008; Yoshinaga et al., 2010). Caterpillar regurgitant can also suppress plant defenses (De Lange et al., 2020). The role of caterpillar regurgitant and plant defenses vary from species to species of plants and insects (Ray et al., 2015; Acevedo et al., 2018; Guo et al., 2020).

Natural variation could be used for dissecting various traits which could be integrated into a crop improvement program. Recently, a nested association mapping (NAM) population for sorghum has been developed by crossing globally diverse lines with a reference line, RTx430, which provides an excellent opportunity to study various valuable agronomic traits (Bouchet et al., 2017). These NAM parental lines have been tested against piercing-sucking insect, greenbugs and sugarcane aphids (Grover et al., 2019; Grover et al., 2020; Grover et al., 2022b), but no information is available on chewing insects. The purpose of this study was to screen NAM parental lines and to dissect the role of phenylpropanoid pathway metabolites in providing resistance/susceptibility to FAW using several different approaches.

## Material and methods

### Plant growth conditions and insects

Ten founder lines of sorghum NAM populations were obtained from USDA-GRIN global germplasm (Beltsville, MD, USA). These were Ajabsido, Macia, P898012, SC35, SC265, SC283, SC971, SC1103, SC1345, and Segaolane, along with the reference line RTx430. The tan/purple lines have been described previously (Funnell-Harris et al., 2013). *HCT*-overexpression (OE) lines were generated as described previously (Walker et al., 2013). The *35S::SbHCT* cassette in the pZP211 binary vector was transformed into grain *S. bicolor* (RTx430) using *Agrobacterium tumefaciens*. Seven independent transgenic events were generated and p-hydroxycinnamoyltransferase (*HCT*) expression (T3 generation) quantified *via* RT-qPCR following methods in Scully et al. (2016). Two homozygous transgenic lines, referred as *SbHCT* ZG-269-1-5a (*HCT* OE1) and *SbHCT* ZG-269-1-11a (*HCT* OE2), were selected based on protein accumulation. ZG-269-1-5a and ZG-269-1-11a were the abbreviations used by the technical staff that conducted transformation experiments. All sorghum plants were grown in 3.8 cm x 21.0 cm plastic Cone-tainers (Hummert International, Earth City, MO) filled with a mix of vermiculite and perlite (PRO-MIX BX BIOFUNGICIDE + MYCORRHIZAE, Premier Tech Horticulture Ltd., Canada) in a greenhouse with a 16-h-light/8-h-dark photoperiod, 25°C, and 50- 60% relative humidity at the University of Nebraska-Lincoln. Plants were watered regularly and fertigated when needed. Sorghum plants at the 3-leaf stage (two to three-week old plants) were used for all the experiments. FAW larvae were obtained from Benzon Research Inc. (Carlisle, PA) and reared on artificial diet in a growth chamber with 16-h-light/8-h-dark photoperiod, 23°C, and 50-60% relative humidity.

### Insect bioassays and sample collection

To screen sorghum NAM parental lines for resistance against FAW, single neonate FAW larvae were released on each sorghum genotype and plants were confined with tubular plastic cages (4 cm diameter by 46 cm height) with vents covered with organdy fabric. All plants were randomly arranged in the plastic racks. The weight of each caterpillar was recorded at 10 days post infestation (dpi). FAW fed leaf areas and undamaged plants were used as FAW infested and control samples, respectively. Leaf samples (~100 mg) were collected for gene expression and metabolomics studies at different time points and immediately flash frozen in liquid nitrogen and stored at -80°C until further use.

### Field evaluation of identified NAM lines for FAW resistance

From the greenhouse screen of the NAM founder lines, we identified SC1345 and Ajabsido as most resistant and susceptible line against FAW compared to RTx430. These genotypes were planted in a field at the University of Nebraska Lincoln farm (Havelock, NE) in 2018 and 2019. In the field, there were four rows (~12.5 feet) in each plot (for each genotype) and each treatment had 4 replications. This whole experiment was surrounded by 4 rows of buffer sorghum plants. At V7-V8 stage of plant, plants were infested with FAW egg masses on each plant. In a single plot, only 5-6 plants grown in the middle were infested with FAW, to avoid crawling of caterpillars to adjacent plots. After 12 days, 10-15 caterpillars were collected to record their weight from each plot.

### FAW regurgitant collection and application to plants

Regurgitant was collected from the oral cavity of fifth-instar artificial diet-fed caterpillars using a micro-pipette and immediately placed on ice. The regurgitant was further diluted 10X into milliQ water. The first and third leaf from the top of SC1345 plants were mechanically wounded using the wounding tool (Ray et al., 2015) and 20 µL of diluted regurgitant applied to each wound every 24 hours for four days. Undamaged plants and wounded plants with water application served as controls. The samples were collected and flash frozen in liquid nitrogen after 4 days of continuous regurgitant application. Three biological replicates of each treatment were collected, and each biological replicate was pooled from three technical replicates.

### RNA extraction and reverse transcription-quantitative PCR

Leaf tissues (80-100 mg) were ground using 2010 Geno/Grinder^®^ (SPEX SamplePrep, NJ, USA) for 40 sec at 1400 strokes/min. The homogenized leaf tissue was added to 1 ml of Sigma-Aldrich TRI reagent (St. Louis, MO, USA). RNA was recovered and purified using the RNA Clean and Concentrator Kit (Zymo Research, Irvine, CA) and on-column DNase treatment was performed. Extracted total RNA was quantified using a Nanodrop 2000c Spectrophotometer (Thermo Scientific™). Complementary DNAs (cDNAs) were synthesized from 1 μg of total RNA using the High-Capacity cDNA reverse transcriptase kit (Applied Biosystems Inc., Foster City, CA). cDNAs were diluted to 1:10 before using them for RT-qPCR. The gene-specific primers used in this study are listed in **Supplemental Table S1**. The RT-qPCR was performed with iTaq™ Universal SYBR^®^ Green Supermix (Bio-Rad Laboratories., Hercules, CA) on a StepOnePlus Real-Time PCR System (Applied Biosystems Inc., Foster City, CA). Three to four independent biological replicates each with three technical replicates were used for RT-qPCR. Relative gene expression of transcripts was analyzed using 2^-ΔΔCT^ method (Livak and Schmittgen, 2001). The mRNA levels were normalized using the internal control *tubulin.* Fold change was calculated by comparing the normalized transcript level of target gene in resistant and susceptible lines to RTx430 (control).

### Metabolite quantification

For metabolomics, leaf samples were collected from RTx430, SC1345, and Ajabsido plants at 10 dpi. The soluble and cell-wall bound phenolic intermediates of the monolignol biosynthesis pathway were quantified on GC-MS as described previously (Scully et al., 2016).

For flavonoids and related compounds, an aliquot of 50-100 mg of samples was extracted using cold methanol:acetonitrile (50:50, v/v) and the tissue samples were disrupted and homogenized by adding two stainless steel beads (SSB 32) using the TissueLyserII (Qiagen) at 10 Hz for 15 mins. After centrifugation at 16,000 x g, the supernatants were collected, and the same extraction was repeated on the pellet one more time. The supernatants were pooled and vacuum dried using a SAVANT speed-vac. The pellets were re-dissolved in 100 μL of 30% methanol. A ZORBAX Eclipse XDB C18 column (2.1 mm × 100 mm, Agilent) on a Shimadzu UHPLC Nexera II was used flowing at 0.4 mL/min. The gradient of the mobile phases A (2% acetic acid) and B (100% acetonitrile) was as follow: 6% B for 1 min, to 17% B in 4 min, to 20% B in 3 min, to 90% B in 8 min, hold at 90% B for 2 min, to 6% B in 1 min. The Shimadzu LC system was interfaced with a Sciex QTRAP 6500+ mass spectrometer equipped with a TurboIonSpray (TIS) electrospray ion source. Analyst software (version 1.6.3) was used to control sample acquisition and data analysis. The QTRAP 6500+ mass spectrometer was tuned and calibrated according to the manufacturer’s recommendations. The flavonoid compounds were detected using MRM transitions that were optimized using standards. For quantification, an external standard curve was prepared using a series of standard samples containing different concentrations of compounds and fixed concentrations of the internal standards mixture. The following compounds were included in the assay: apigenin, catechin, chalconaringenin, cyanidin, daidzein, delphinidin, epicatechin, genistein, hesperetin, kaempferol, luteolin, naringenin, phloretin, proanthocyanindin A2, procyanidin B2, quercetin, quercetin-3-galactoside, quercetin-3-glucoside, resveratrol, and rutin. Total flavonoids was estimated using spectrophotometer as described previously (Tetreault et al., 2021).

### Lignin quantification

The lignin was quantified using the thioglycolic acid method as described previously (Moreira-Vilar et al., 2014; Kundu et al., 2018).

### Statistical analyses

Statistical analyses were performed using PROC GLIMMIX in SAS 9.4 (SAS Institute, Cary, NC). Normality and homogeneity of data were tested using graphical analysis of all residuals and Shapiro-Wilk test, respectively. To evaluate the effect of genotypes on insect performance, one-way analysis of variance (ANOVA) was used. Pairwise comparisons between treatments were carried out by comparing the means with Tukey’s honestly significant difference tests (*P* < 0.05).

## Results

### SC1345 displayed enhanced resistance against FAW under greenhouse and field conditions

**Figure 1:**
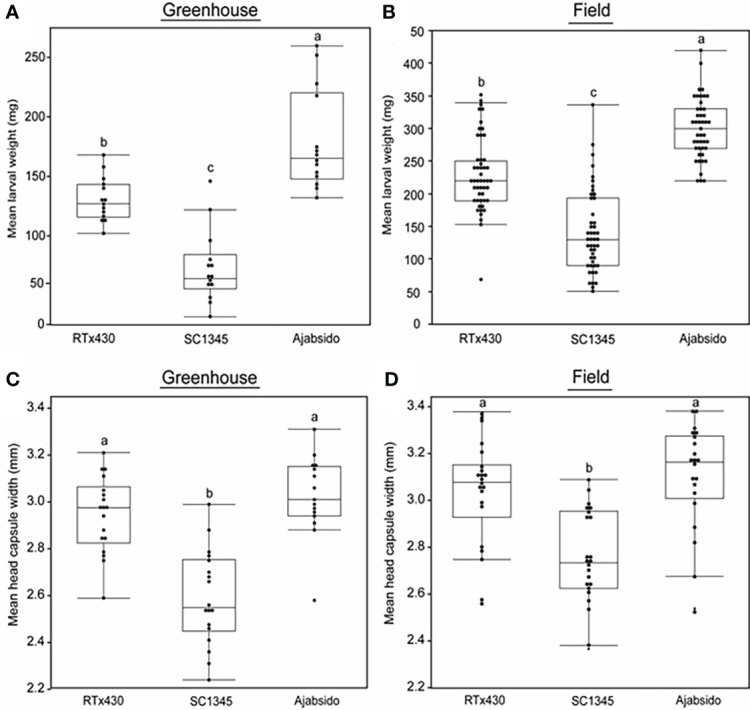
SC1345 provided enhanced resistance to fall armyworm (FAW). **(A)** Mean larval weight of FAW collected from sorghum lines, 10 days after the introduction of newly hatched larvae on sorghum (three-leaf stage) plants under greenhouse conditions (n = 14). **(B)** Mean larval weight of FAW collected from sorghum lines, 12-14 days after the FAW egg masses infestation on sorghum (nine-leaf stage) plants under field conditions. Data from 2018 and 2019 field experiments were pooled to represent mean data (n = 45-49). **(C)** Mean head capsule width of FAW caterpillars collected from sorghum lines, 10 days after the introduction of newly hatched larvae on sorghum (three-leaf stage) plants under greenhouse conditions (n = 15-18). **(D)** Mean head capsule width of FAW caterpillars collected from sorghum lines, 12-14 days after infestation on sorghum (nine-leaf stage) plants under field conditions in 2019 (n = 21-22). Error bars represent ± SE. Different letters indicate significant difference relative to each other (*P* < 0.05).

NAM populations provide a unique platform to study genetic diversity of crops and to dissect complex traits (Poland et al., 2011; Bajgain et al., 2016; Vatter et al., 2017). To explore the natural variation in sorghum for resistance to FAW, we screened NAM parental lines along with control RTx430 by performing a no-choice assay (**Supplementary Figure S1**). Results indicated that FAW gained significantly least weight on SC1345 and highest weight on Ajabsido compared to control, RTx430 plants (**Figures 1A**, **S1**). We confirmed the resistance status of SC1345 and Ajabsido in comparison to RTx430 under field conditions as well, and a similar pattern was observed in 2018 and 2019 (**Figure 1B**). We measured the head capsule width of caterpillars collected from RTx430, SC1345, and Ajabsido plants at 10 dpi. FAW collected from SC1345 plants displayed significantly lesser head capsule width compared to larvae collected from RTx430 and Ajabsido plants under both greenhouse and field conditions (**Figures 1C, D**, **S2**). No significant difference between head capsule width of caterpillars collected from RTx430 and Ajabsido were found. These results suggest that resistance and susceptible traits in NAM founder lines could be used for understanding the underlying resistance mechanisms in sorghum to FAW. For further experiments, the most FAW resistant and susceptible lines (SC1345 and Ajabsido), along with the reference line, RTx430 were used (**Supplementary Figure S3**).

### FAW infestation altered the expression level of genes involved in monolignol biosynthetic pathway

**Figure 2:**
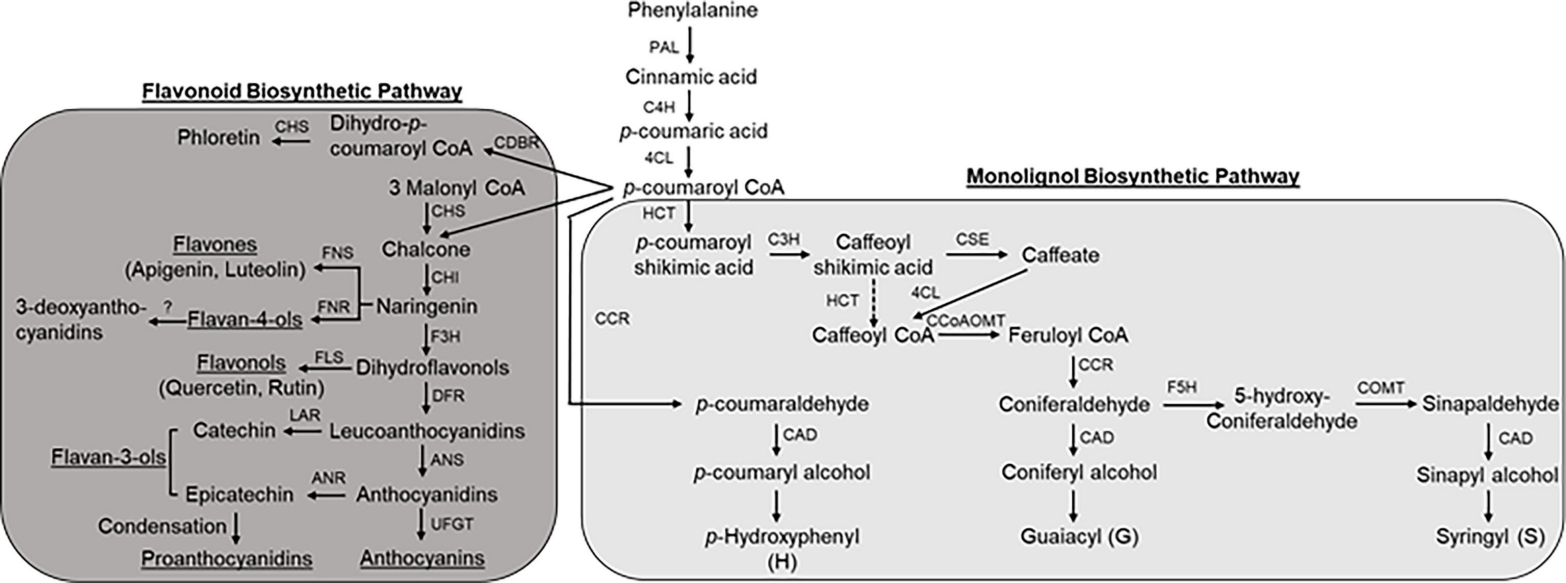
Schematic representation of phenylpropanoid pathway in sorghum (modified from Scully et al., 2016). Enzyme abbreviations: PAL, phenylalanine ammonia lyase; C4H, cinnamate 4-hydroxylase; 4CL, 4-coumarate:CoA ligase; HCT, *p*-hydroxycinnamoyltransferase; C3H, 4-coumarate hydroxylase; CSE, caffeoyl shikimate esterase; CCoAOMT, caffeoyl-CoA-*O*-methyltransferase; CCR, cinnamoyl-CoA reductase; F5H, ferulate 5-hydroxylase; COMT, caffeic acid *O*-methyl transferase; CAD, cinnamyl alcohol dehydrogenase; CHS, chalcone synthase; CHI, chalcone isomerase; FNS, flavone synthase; F3H, flavanone 3-hydroxylase; FLS, flavonol synthasae; DFR, dihydroflavonol 4-reductase; LAR, leucoanthocyanidin reductase; ANS, anthocyanidin synthase; ANR, anthocyanidin reductase; UFGT, UDP-glucose: flavonoid 3-O-glucosyltransferase.

The induction of monolignol biosynthetic pathway genes has been well correlated with resistance to stress conditions such as pathogens and water stress (Moura et al., 2010). To determine a role of the monolignol biosynthetic pathway in sorghum resistance to FAW infestation, the expression levels of several genes (*PAL*, phenylalanine ammonia lyase; *C4H*, cinnamate 4-hydroxylase; *4CL*, 4-coumarate:CoA ligase; *HCT*, p-hydroxycinnamoyltransferase; *C3H*, 4-coumarate hydroxylase; *CSE*, caffeoyl shikimate esterase; *CCoAOMT*, caffeoyl-CoA-O-methyltransferase; *CCR*, cinnamoyl-CoA reductase; *F5H*, ferulate 5-hydroxylase; *COMT*, caffeic acid O-methyl transferase; *CAD*, cinnamyl alcohol dehydrogenase; **Figure 2**; **Table S1**) involved in monolignol biosynthesis were evaluated after 10 dpi (**Supplementary Figure S4**). No significant changes in expression were observed for *PAL* and *F5H* for any comparisons. In RTx430 plants infested with FAW, significant changes in gene expression relative to control uninfested plants were seen only for *CAD* (**Supplementary Figure S4**). In the resistant SC1345 plants, five genes, namely, *4CL, HCT, CCoAOMT, CCR, and COMT*, were downregulated after FAW infestation, whereas *C4H* and *CAD* were strongly upregulated in the resistant line (**Supplementary Figure S4**). For FAW infested susceptible Ajabsido plants, *HCT* and *CCR* expression were significantly downregulated, whereas *C4H, 4CL*, and *CCoAOMT* were significantly upregulated (**Supplementary Figure S4**).

### FAW infestation suppressed soluble phenolic compounds in sorghum

**Figure 3:**
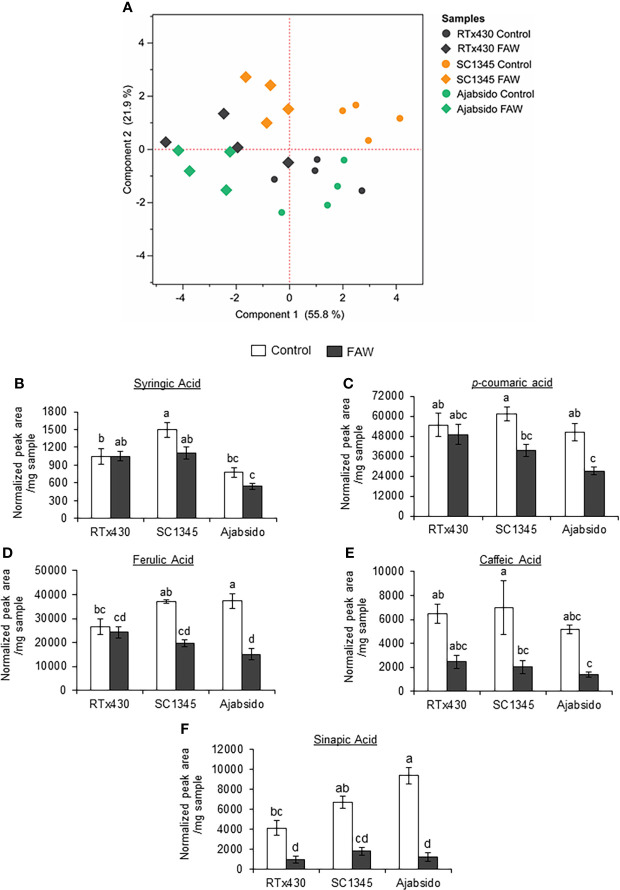
Fall armyworm (FAW) feeding suppressed the accumulation of phenolic intermediates of the monolignol biosynthetic pathway in sorghum. **(A)** Principal component analysis (PCA) of soluble and cell-wall bound phenolic compounds quantified by GC-MS after 10 days of FAW infestation on RTx430, SC1345 and Ajabsido plants. FAW-uninfested plants were used as controls. **(B–F)** Relative abundances of intermediates of the monolignol biosynthetic pathway were determined by GC/MS analysis of soluble phenolics extracted from sorghum leaves. Peak area was normalized to the internal standards using 4-methyl cinnamic acid for soluble phenolics. The relative abundances of soluble **(B)** syringic acid, **(C)** *p*-coumaric acid, **(D)** ferulic acid, **(E)** caffeic acid, and **(F)** sinapic acid are presented (n = 4). Error bars represent ± SE. Different letters indicate significant difference relative to each other (*P* < 0.05).

To determine how FAW infestation affected the metabolic changes through the monolignol biosynthetic pathway, plant samples were evaluated for soluble and cell wall bound phenolic compounds by GC-MS at 10 dpi. Principal Component Analysis (PCA) of phenolic compounds was performed (**Figure 3A**). PC1 accounting for 55.8% of the variance separated the samples obtained from controls from the FAW-infested plants. PC2 accounting for 21.9% of the variance, essentially separated the FAW-infested Ajabsido from the SC1345 plants. Notably, PC2 also separated the control SC1345 plants from Ajabsido and RTx430 control plants (**Figure 3A**). Although there was no change in the levels of syringic acid before and after FAW infestation on all three sorghum lines tested (**Figure 3B**), our results suggest that FAW infestation suppressed the accumulation of *p*-coumaric acid, ferulic acid, and caffeic acid in SC1345 and Ajabsido plants, but not in RTx430 plants (**Figures 3C–E**). The sinapic acid levels were significantly reduced upon FAW infestation for all three sorghum lines tested as compared to control uninfested plants (**Figure 3F**). Significant changes in wall bound phenolics were not observed before or after FAW attack (**Supplementary Figure S5**). Similarly, no changes in lignin content determined by the thioglycolic acid method were observed between the FAW resistant (SC1345) and susceptible (Ajabsido) plants before or after FAW feeding (**Supplementary Figure S6**). These results indicated that plants might divert monolignol biosynthesis intermediates to other pathways in order to provide resistance to FAW.

### SC1345 showed upregulation of the genes involved in flavonoid pathway upon FAW infestation

To understand a potential diversion of intermediates from the monolignol biosynthetic pathway to the flavonoid pathway, we performed RT-qPCR with three candidate genes encoding flavone synthase II (*FNSII*), flavanone 4-reductase (*FNR*), and chalcone synthase (*CHS*) (**Table S1**) that are important for the synthesis of flavonoids in plants (Dao et al., 2011; Mizuno et al., 2016; Wang et al., 2020). We found significantly higher elevated levels of *FNSII* and *FNR* genes only in FAW-infested SC1345 plants compared to uninfested SC1345 plants at 10 dpi (**Supplementary Figure S7**). Because CHS supplies substrates for the other two enzymes that were strongly upregulated only in FAW-infested SC1345 plants, we evaluated if flavonoid content was altered as well.

### SC1345 showed elevated levels of several flavonoid compounds after FAW attack

**Figure 4:**
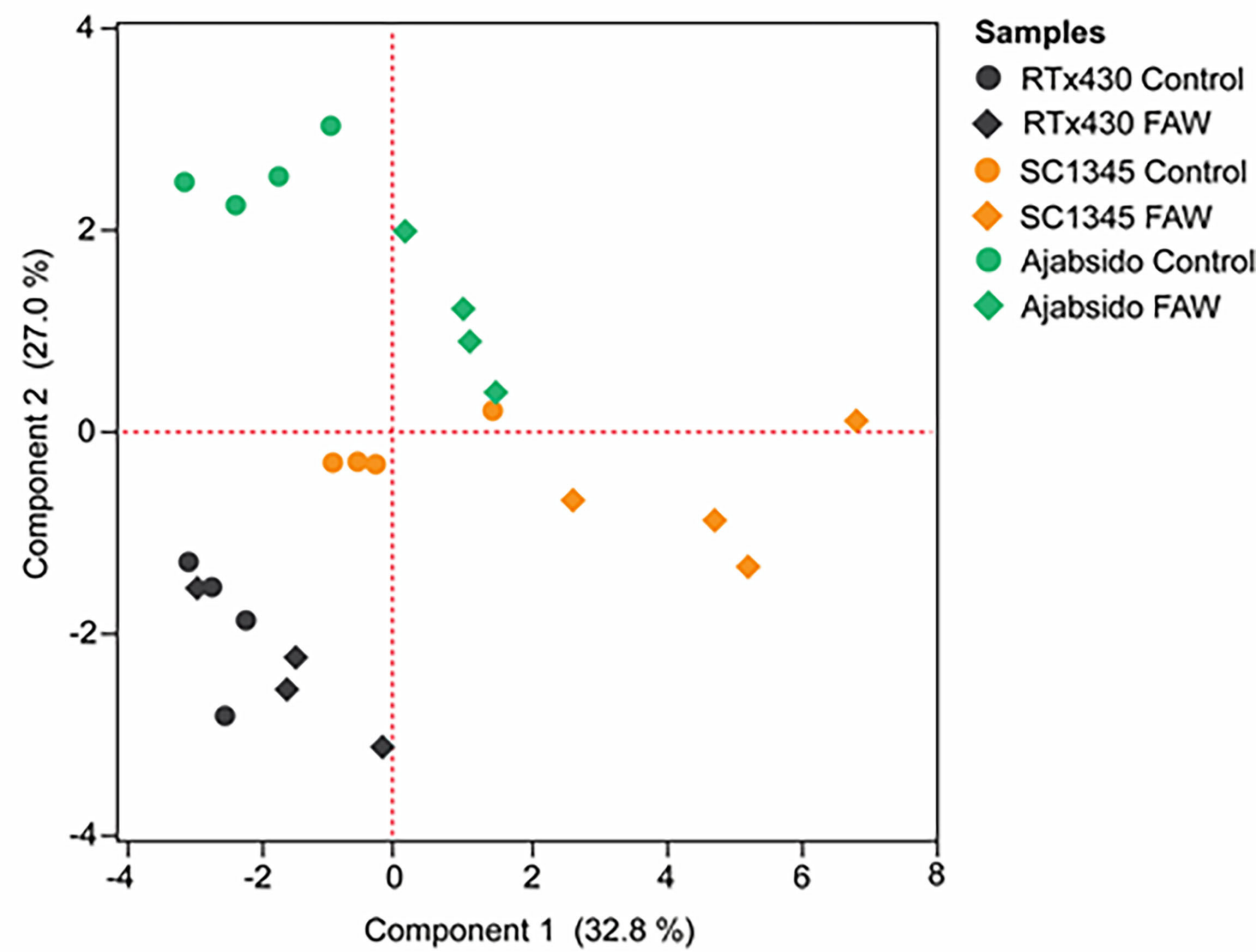
Principal component analysis (PCA) of flavonoid compounds quantified after 10 days of fall armyworm (FAW) infestation on RTx430, SC1345 and Ajabsido plants. FAW-uninfested plants were used as controls.

**Figure 5:**
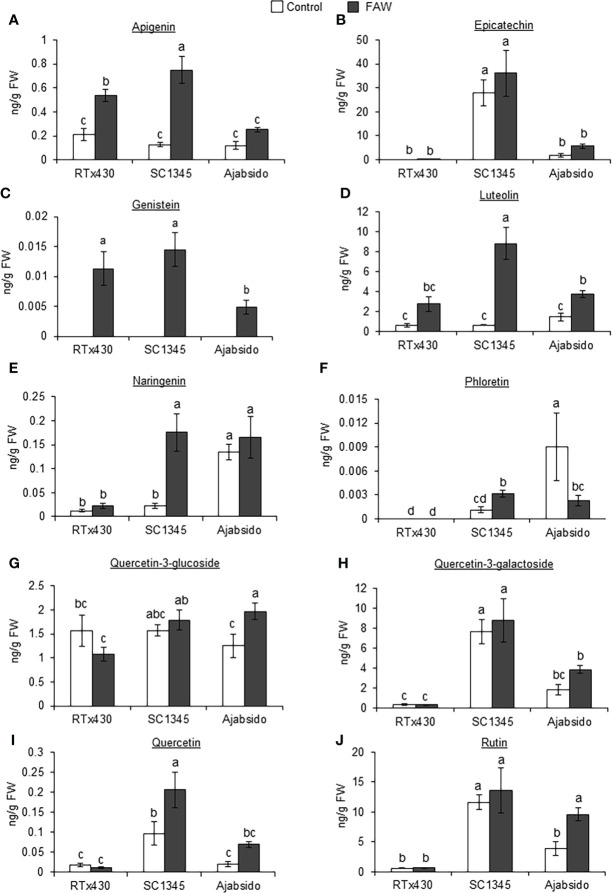
Fall armyworm (FAW) infestation enhanced the levels of several flavonoid compounds in SC1345 plants. Absolute abundances of flavonoid compounds, **(A)** Apigenin, **(B)** Epicatechin, **(C)** Genistein, **(D)** Luteolin, **(E)** Naringenin, **(F)** Phloretin, **(G)** Quercetin-3-glucoside, **(H)** Quercetin-3-galactoside, **(I)** Quercetin, and **(J)** Rutin, in RTx430, SC1345, and Ajabsido plants before and after FAW infestation for 10 days (n = 3-4). FW, fresh weight. Error bars represent ± SE. Different letters indicate significant difference relative to each other (*P* < 0.05).

**Figure 6:**
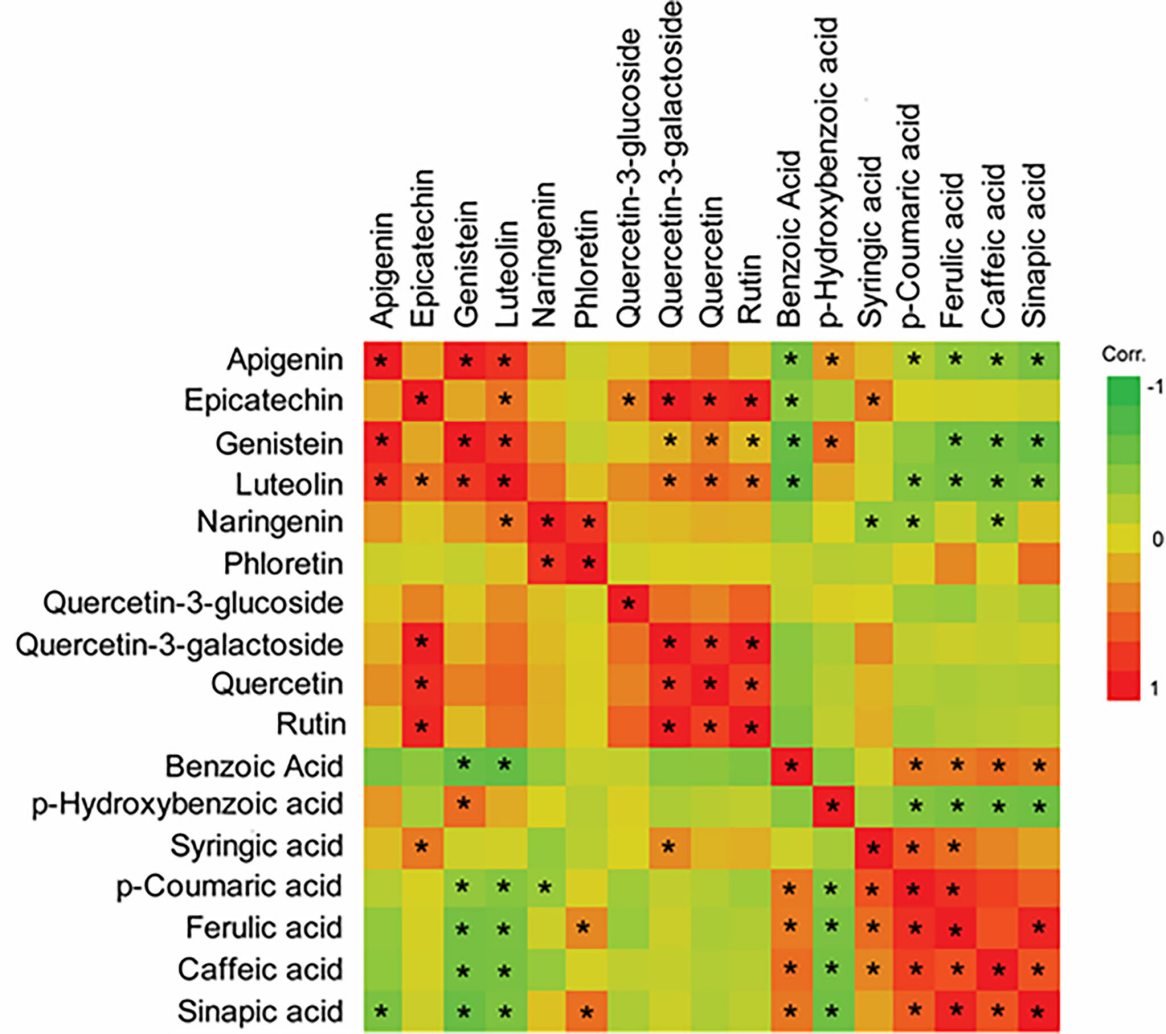
Correlation plot of phenolics and flavonoid compounds differentially changed after 10 days of fall armyworm (FAW) infestation on sorghum plants. A Pearson pairwise correlation was calculated for differentially induced phenolic and flavonoid compounds in JMP Pro 14. Asterisks are used to show significant correlations between compounds (*P* < 0.05).

Flavonoid compounds in RTx430, SC1345, and Ajabsido leaf tissues collected after 10 days of FAW infestation were quantitated by HPLC. PCA of detected flavonoid compounds showed that components 1 and 2 accounted for0 59.8% of the total variance and PC1 mainly contributes to clustering of SC1345 and Ajabsido plants after FAW attack, but not of the RTx430 plants (**Figure 4**). Out of all the detectable flavonoids compounds, there were 10 flavonoid compounds with significantly elevated levels in at least one of the three lines after FAW attack (**Figure 5**). Out of these 10 compounds, seven flavonoids were significantly higher either constitutively or induced after FAW infestation compared to their respective FAW-uninfested control plants (**Figure 5**). Levels of apigenin, luteolin, and quercetin were significantly increased in SC1345 plants after FAW infestation compared to RTx430 and Ajabsido lines (**Figures 5A, D, I**). Genistein was only detected upon FAW infestation, and its levels were significantly lower in Ajabsido plants compared to the two other lines (**Figure 5C**). We found a significant increase in the levels of naringenin and phloretin in SC1345 plants after FAW infestation compared to RTx430 and Ajabsido plants (**Figures 5E, F**). No significant differences in the levels of quercetin-3-glucoside were observed before and after FAW infestation in RTx430 and SC1345 plants, however, FAW infestation for 10 days increased the level of quercetin-3-glucoside in Ajabsido plants (**Figure 5G**). Our results also indicate that the epicatechin, quercetin-3-galactoside, and rutin levels were constitutively higher in SC1345 plants, compared to RTx430 and Ajabsido plants (**Figures 5B, H, J**). Furthermore, a correlation plot between phenolics and flavonoid compounds showed mainly negative correlation between their accumulation in plants (**Figure 6**). These data suggest that SC1345 redirects the metabolite synthesis towards the flavonoid pathway after FAW infestation.

### Sorghum flavonoids are crucial for providing resistance to FAW

**Figure 7:**
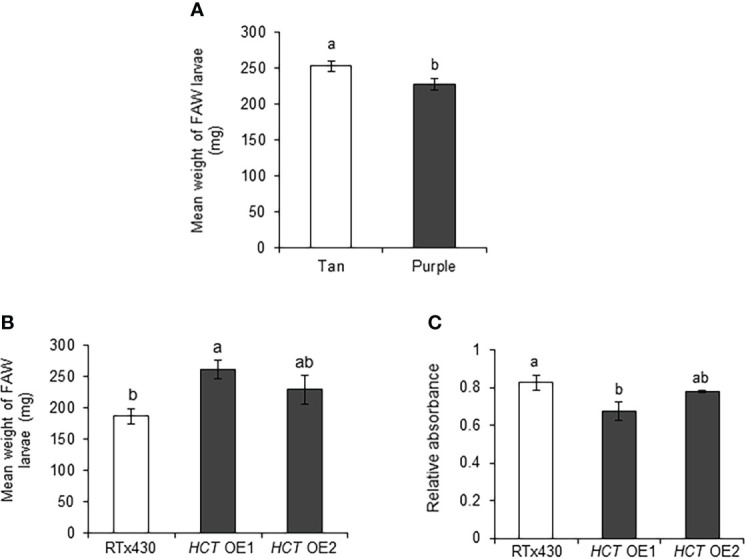
Sorghum flavonoids affected the fall armyworm (FAW) growth adversely. Mean larval weight of FAW collected from **(A)** sorghum tan and purple lines (n = 11-15 for each sibling line, total n = 63-79), and **(B)** RTx430 (wild-type) and *HCT* (*p*-hydroxycinnamoyltransferase)-overexpression (OE) events (n = 19-21), 10 days after the introduction of newly hatched larvae on sorghum (three-leaf stage) plants under greenhouse conditions. **(C)** Total flavonoids estimated using spectrophotometer on two-week-old RTx430 and *HCT* OE plants (n = 3-4). Error bars represent ± SE. Different letters indicate significant difference relative to each other (*P* < 0.05).

Previously, near-isogenic sorghum lines were developed that exhibit different plant colors, tan and purple lines of sorghum (Funnell and Pedersen, 2006; Funnell-Harris et al., 2013). Tan plants lack the ability to synthesize flavonoid pigments while purple plants maintain this ability. We utilized these lines to observe the effects of flavonoids on FAW. We found that FAW weight was significantly higher on tan plants, compared to purple plants (**Figure 7A**). Moreover, it has been shown that *HCT*-silenced Arabidopsis plants displayed redirecting the metabolic flux from lignin pathway into flavonoids (Besseau et al., 2007). In this study, we used sorghum *HCT*-overexpression lines to study the effect of reduced flavonoids on FAW growth. As expected, we found that FAW weight was significantly higher in an *HCT* OE1 event (**Figure 7B**). Furthermore, *HCT* OE1 line had significantly lower flavonoid levels than RTx430 plants (**Figure 7C**). These multiple lines of evidence suggest that flavonoids provide resistance to FAW in sorghum.

### FAW regurgitant elevated the expression levels of genes involved in sorghum flavonoid pathway

**Figure 8:**
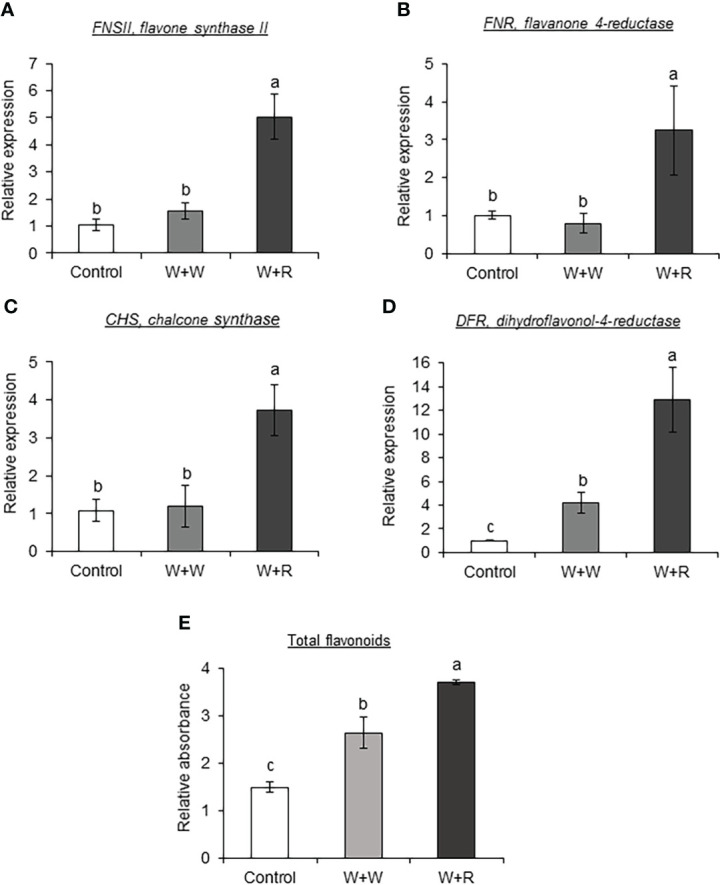
Application of fall armyworm (FAW) regurgitant on sorghum wounded plants enhanced the expression levels of flavonoid pathway genes. RT-qPCR analysis of flavonoid pathway genes **(A)** *FNSII*, flavone synthase II; **(B)** *FNR*, flavanone 4-reductase; **(C)** *CHS*, chalcone synthase; and **(D)** *DFR3*, Dihydroflavonol-4-reductase. **(E)** Total flavonoids estimated using spectrophotometer in leaves of sorghum SC1345 plants after four applications of FAW regurgitant every 24 hours. Undamaged and wounded plants were used as controls (n = 3-4). Error bars represent ± SE. Different alphabets indicate significant difference relative to each other (*P* < 0.05). W + W, treatments in which water was applied to wounds; W + R, treatments in which FAW regurgitant was applied to wounds.

To determine the effect of caterpillar regurgitant in regulating the phenylpropanoid-metabolic changes, SC1345 plants were mechanically wounded and treated with regurgitant. Application of FAW regurgitant resulted in significantly higher expression of *FNSII*, *FNR*, *CHS*, and *DFR3* (**Table S1**) genes in SC1345 wounded plants after 4 days of regurgitant application (**Figures 8A–D**). On the other hand, FAW regurgitant application on SC1345 wounded plants did not impact the expression levels of genes involved in the monolignol biosynthetic pathway (**Supplementary Figure S8**). The total flavonoid estimation using spectrophotometric approach confirmed the gene expression results (**Figure 8E**).

## Discussion

Our results indicated that the sorghum genotypes SC1345 and Ajabsido were the most resistant and susceptible lines, respectively, to FAW among the NAM parental lines tested under both greenhouse and field conditions compared to RTx430 plants. Metabolite, gene-expression, FAW growth on altered levels of flavonoid sorghum plants, and caterpillar regurgitant application to SC1345 plants, provided good evidence for a defensive role for flavonoids in the FAW-resistant genotype. Previously, we have shown that SC1345 plants supported higher number of aphids compared to RTx430 plants and also exhibited a weakened photosynthetic machinery to handle aphid stress (Grover et al., 2020). These results suggested that SC1345 plants had the innate ability in allocating resources towards secondary metabolites, which could potentially attenuate the primary defense mechanisms against insect pests and investing lesser resources towards plant growth. Lignin accumulation and changes in lignin pathway associated-metabolites have been correlated with altered defense responses of plants to insects (Bernards and Båstrup-Spohr, 2008; Gallego-Giraldo et al., 2011; Santiago et al., 2013; Gallego-Giraldo et al., 2018). In addition to the production of monolignols needed for lignin biosynthesis, the phenylpropanoid pathway generates several phenolic compounds that have important defensive roles in plants and have been associated with resistance to insects (Dreyer and Jones, 1981; McPherson et al., 2014; Dixit et al., 2017). It has also been suggested that modulations in phenylpropanoid genes do not necessarily implicate lignin biosynthesis, but the pathway intermediates may have functions related to antioxidants/defenses (Cesarino, 2019). In fact, our results suggest that there was no change in lignin accumulation in SC1345 plants compared to Ajabsido plants (**Supplementary Figure S6**), indicating that the pathway intermediates may be diverted to the production of a range of secondary compounds, such as flavonoids.

Data from lignin biosynthetic mutants in sorghum, and other plants indicate varying degrees of diversion of metabolism away from the biosynthesis of monolignols, and lignin to other related pathways (Gallego-Giraldo et al., 2018; Saluja et al., 2021). We found the downregulation of several genes involved in lignin biosynthesis pathway after 10 days of FAW infestation in SC1345 plants (**Supplementary Figure S4**). Several phenolics such as quercetin, gallic acid, caffeic acid, syringic acid, *p*-coumaric acid, ferulic acid, and cinnamic acid showed herbicidal and insecticidal activities against duckweed, *Lemna minor* L., and the dusky cotton bug, *Oxycarenus hyalinipennis*, respectively (Younus et al., 2021). Similarly, phenolic compounds resveratrol and *p*-coumaric acid have been reported to reduce larval weights of *S. litura* and *Amsacta albistriga* (Sambangi and Rani, 2016). In cotton, chewing insects, *Helicoverpa armigera* and *S. litura*, induced accumulation of gallic acid, 4-cinnamic acid, *p*-coumaric acid, and salicylic acid in leaves. All of these phenolics caused significant weight reduction and mortalities in target insects (Dixit et al., 2017). FAW has been shown to have reduced growth when phenolic acids (ferulic, vanillic, sinapic, and syringic acids) were incorporated in artificial diets (Dowd and Sattler, 2015). Surprisingly, we found suppression of levels of soluble phenolic compounds in all three genotypes tested, irrespective of the resistance status, again suggestive of formation of other defensive compounds. It is plausible that the changes in patterns of accumulation of phenolic compounds after FAW infestation attribute to the diverse genetic makeup of the plants and possibly leading to different functions and phenotypes.

In the current study, we found elevated levels of flavonoids in SC1345 plants upon FAW attack while phenolics accumulation was suppressed. Moreover, sorghum lines with reduced levels of flavonoids sustained better FAW growth compared to controls, providing more evidence for a defensive role for sorghum flavonoids against FAW. Recently, it was shown that sorghum flavonoids extracted from leaves caused FAW mortality when incorporated in artificial diet (Chatterjee et al., 2022). Furthermore, sorghum 3-deoxyanthocyanidins-sprayed leaves of a susceptible maize line deterred herbivory by challenging the survival of the FAW larvae (Chatterjee et al., 2022). These results also align with our bioassay results of sorghum lines with altered levels of flavonoids.

Chewing insects release cues such as regurgitant and saliva while feeding on plants. These cues could have beneficial or detrimental effects depending on the complex interactions between plants and insects. Previously, FAW regurgitant and saliva was reported to induce defense responses in maize (Chuang et al., 2013). Caterpillar regurgitant has been reported to have several HAMPs such as β-glucosidase, fatty acid-amino acid conjugates (FACs), and inceptins, which can induce plant defenses (Halitschke et al., 2001; Schmelz et al., 2006; Yoshinaga et al., 2008; Yoshinaga et al., 2010). Whereas caterpillar regurgitant of two herbivores, *Pieris brassicae* and *S. littoralis*, has also been shown to suppress plant defenses in Arabidopsis, specifically *via* the hydroperoxide lyase (HPL) branch of oxylipin pathway (Consales et al., 2012; Savchenko et al., 2013). These studies clearly indicate that insect cues could alter specific defense responses and their effects vary in diverse plant-insect systems. Previously, it has been reported that FAW possesses a heat-stable effector in caterpillar regurgitant that has the ability to suppress green leaf volatile emission in maize plants (Jones et al., 2019). In this current study, application of FAW regurgitant induced several genes encoding enzymes needed for flavonoid biosynthesis and enhanced flavonoid content as well but did not impact the expression of genes related to monolignol biosynthetic pathway. These data indicate a robust response of the resistant plants to FAW secretions and point to flavonoids as effective defensive components. Future experiments are needed to understand and tease apart the role of other FAW cues, for example, saliva and frass, in modulating phenylpropanoid pathway metabolites.

Overall, our findings indicate that changes in the utilization of phenylpropanoid pathway intermediates in sorghum could be an important plant defense mechanism against FAW. Increase in flavonoids could underlie the resistance of the SC1345 plants relative to the parental line RTx430 and the susceptible line Ajabsido. The induction of flavonoids by larval regurgitant in SC1345 plants lends further support to this hypothesis. The current study provides the basis to explore the role of other genes, signaling networks, secondary metabolites, and/or regulatory mechanisms that underlie sorghum resistance to FAW. Crop improvement through enhancing innate defense mechanisms using various biotechnological tools, would increase the level and durability of resistance to reduce FAW infestations.

## Data availability statement

The original contributions presented in the study are included in the article/**Supplementary Material**. Further inquiries can be directed to the corresponding author.

## Author contributions

SG and JL conceived and designed the research; SG, SS, HP, and NP performed the research; GS and SES contributed reagents, methods development and provided guidance on experiments; SG and JL wrote the paper. All authors reviewed and edited the manuscript. All authors contributed to the article and approved the submitted version.

## Funding

This work was supported by USDA-NIFA grant # 2020-67013-31857.

## Acknowledgments

We would like to acknowledge John Toy for help with sorghum seed production. We also thank Juan Cardona for greenhouse assistance and the Proteomic and Metabolomics Facility (Center for Biotechnology at the University of Nebraska-Lincoln) for sorghum metabolomic analysis. The authors would also like to thank Drs. Troy Anderson and Ana Maria Vélez (Department of Entomology, University of Nebraska-Lincoln) for providing access to laboratory equipment for some of the experiments performed in this study.

## Conflict of interest

The authors declare that the research was conducted in the absence of any commercial or financial relationships that could be construed as a potential conflict of interest.

The reviewer AB declared a shared affiliation with the authors NP, GS, and SES to the handling editor at the time of review.

## Publisher’s note

All claims expressed in this article are solely those of the authors and do not necessarily represent those of their affiliated organizations, or those of the publisher, the editors and the reviewers. Any product that may be evaluated in this article, or claim that may be made by its manufacturer, is not guaranteed or endorsed by the publisher.
