# Supplementary material for: Dynamic regulation of phenylpropanoid pathway metabolites in modulating sorghum defense against fall armyworm
Source: Front Plant Sci. 2022 Nov 25;13:1019266. doi: 10.3389/fpls.2022.1019266 (PMC9732255; doi:10.3389/fpls.2022.1019266)
Supplement: Supplementary file 1 [file DataSheet_1.pdf]

Supplementary Information

Dynamic regulation of phenylpropanoid pathway metabolites in modulating sorghum defense against fall armyworm

Sajjan Grover[1], Sanket Shinde[1], Heena Puri[1], Nathan Palmer[2], Gautam Sarath[1,2], Scott E. Sattler[2], and Joe Louis[1,3,*]

[1]Department of Entomology, University of Nebraska-Lincoln, Lincoln, NE 68583
[2]Wheat, Sorghum, and Forage Research Unit, U.S. Department of Agriculture-Agricultural Research Service, Lincoln, NE 68583
[3]Department of Biochemistry, University of Nebraska-Lincoln, Lincoln, NE 68583

***Author for Correspondence:** Joe Louis
Department of Entomology and Department of Biochemistry
University of Nebraska-Lincoln, Lincoln, NE 68583, USA
E-mail: joelouis@unl.edu; Phone: +1 (402) 472-8098

Figure S1
Grover et al.

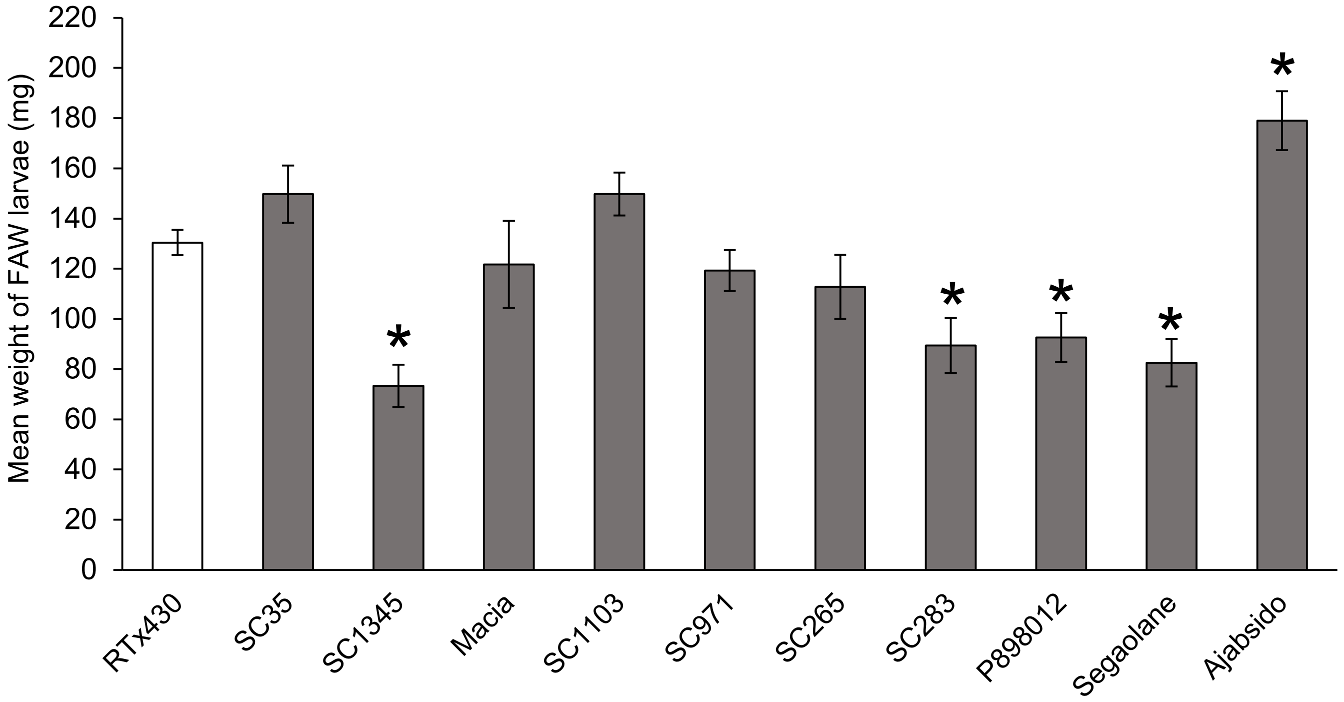

Supp. Fig. S1 Mean larval weight of fall armyworm (FAW) collected from sorghum lines, 10 days after the introduction of newly hatched larvae on sorghum (three-leaf stage) plants under greenhouse conditions (n = 14). Error bars represent ± SE. Asterisks indicate significant difference relative to RTx430 ($P < 0.05$; Dunnett's test).

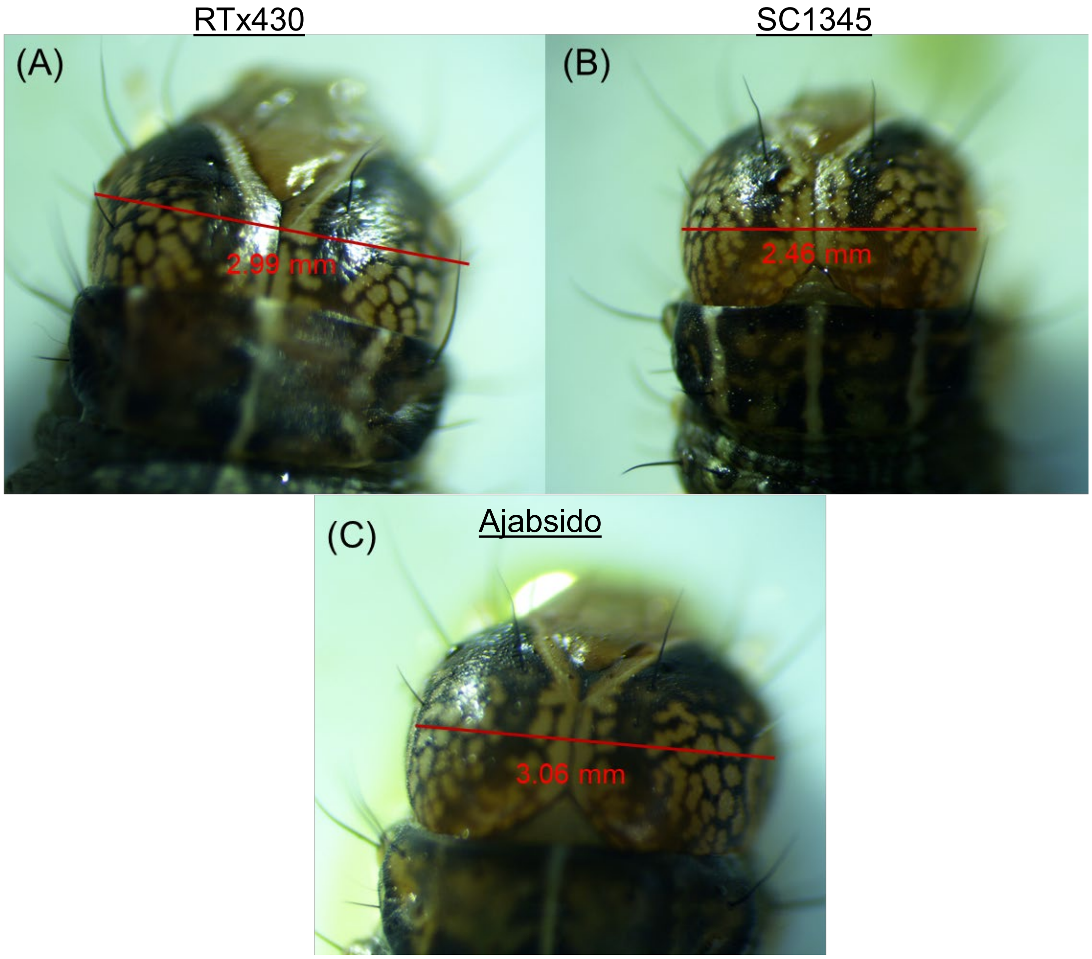

Supp. Fig. S2 Head capsule images of caterpillars collected from RTx430, SC1345 and Ajabsido plants, 10 days after the introduction of newly hatched fall armyworm larvae on sorghum (three-leaf stage) plants under greenhouse conditions.

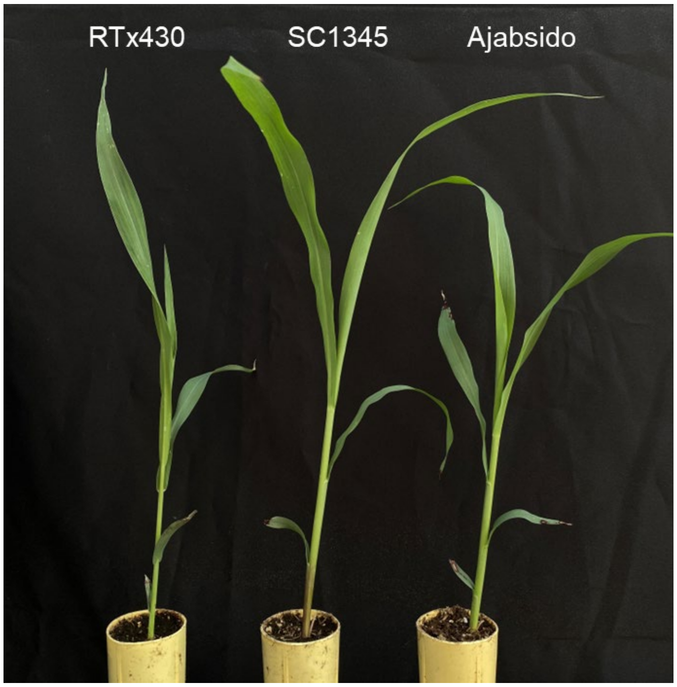

Supp. Fig. S3 Comparison of the morphology of two-week-old RTx430, SC1345 and Ajabsido sorghum plants.

Figure S4
Grover et al.

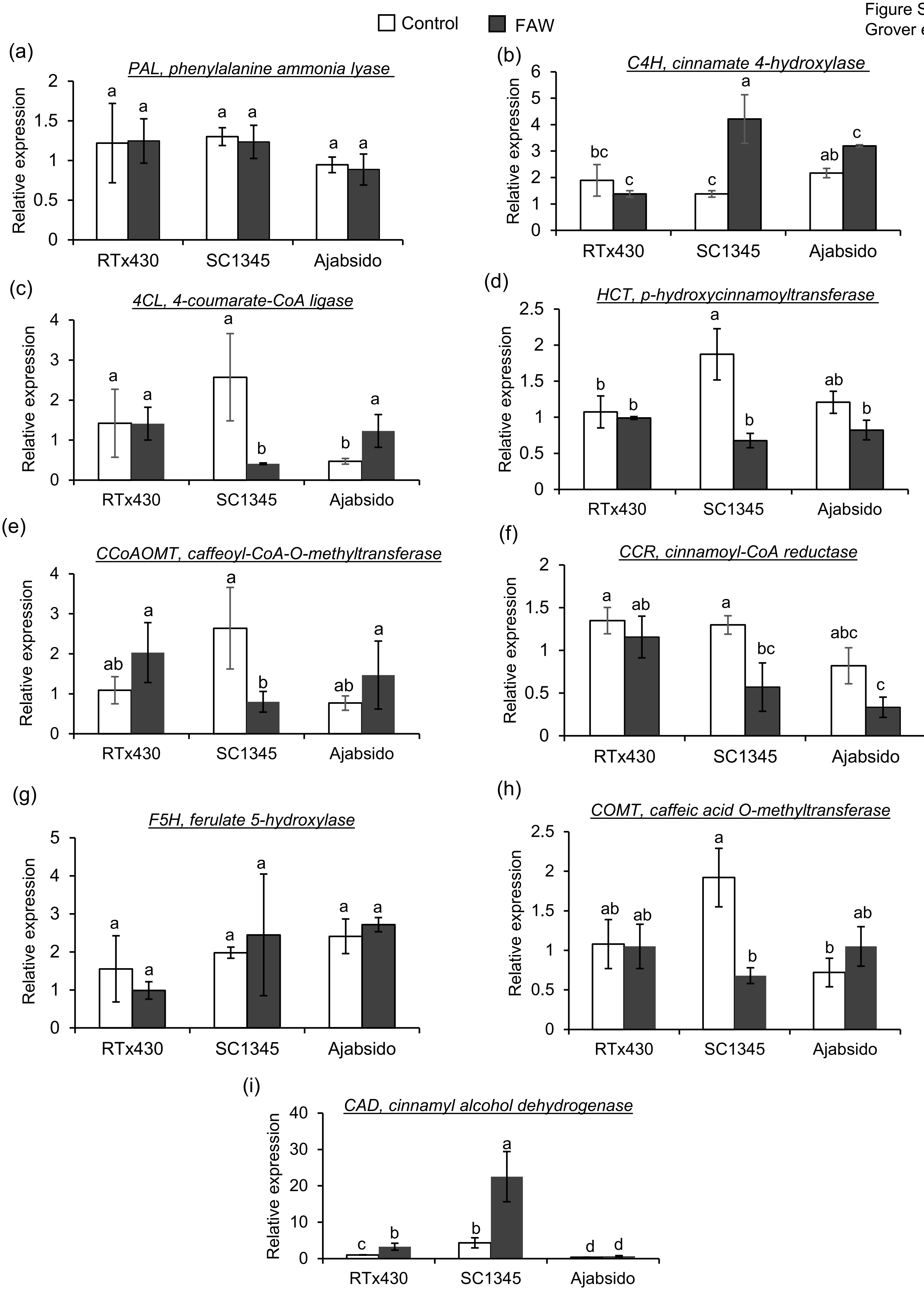

Supp. Fig. S4 Fall armyworm (FAW) feeding alters the expression of genes involved in monolignol biosynthetic pathway in sorghum. RT-qPCR analysis of sorghum lignin pathway genes in sorghum RTx430, Ajabsido, and SC1345 leaves after feeding by FAW larvae for 10 days. (a) *PAL*, phenylalanine ammonia lyase; (b) *C4H*, cinnamate 4-hydroxylase; (c) *4CL*, 4-coumarate: CoA ligase; (d) *HCT*, p-hydroxycinnamoyltransferase; (e) *CCoAOMT*, caffeoyl-CoA-O-methyltransferase; (f) *CCR*, cinnamoyl-CoA reductase; (g) *F5H*, ferulate 5-hydroxylase; (h) *COMT*, caffeic acid O-methyl transferase; and (i) *CAD*, cinnamyl alcohol dehydrogenase. Plants that were not infested with FAW were used as controls (n = 3-4). Error bars represent ± SE. Different letters indicate significant difference relative to each other ($P < 0.05$).

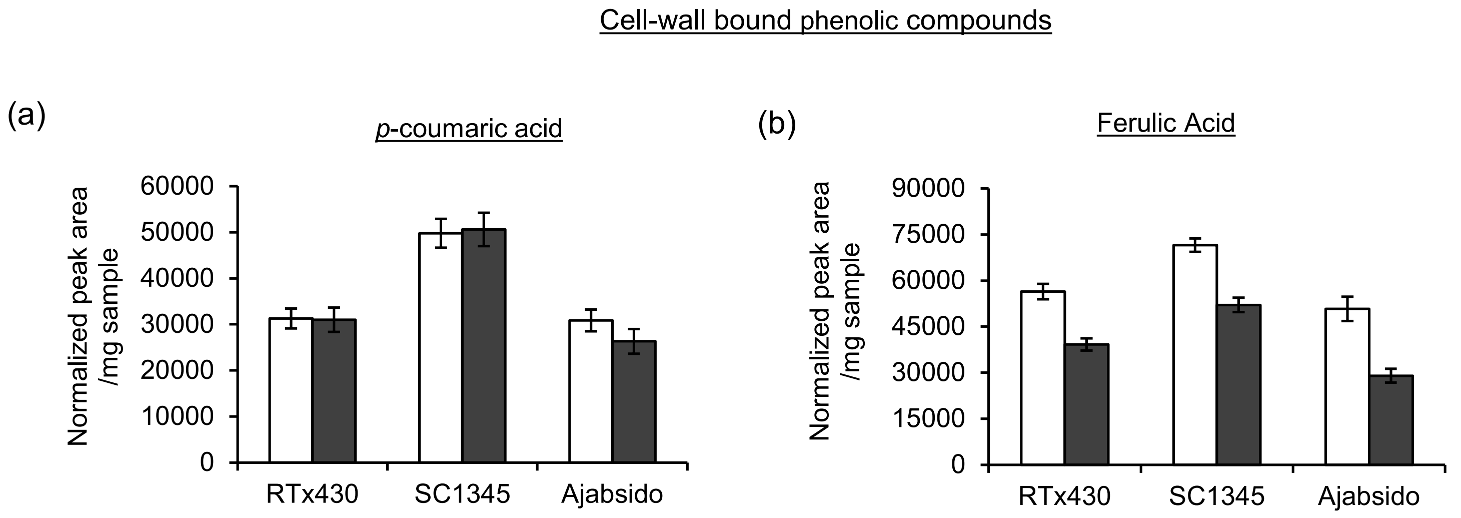

Supp. Fig. S5 Relative abundances of intermediates of the monolignol biosynthetic pathway were determined by GC/MS analysis of cell-wall bound phenolics extracted from sorghum leaves. Peak area was normalized to the internal standard using alpha-methyl cinnamic acid for cell wall-bound phenolics. The relative abundances of wall-bound (a) *p*-coumaric acid and (b) ferulic acid are presented ($n = 4$). Error bars represent ± SE. No significant differences were observed among any of the treatments ($P > 0.05$).

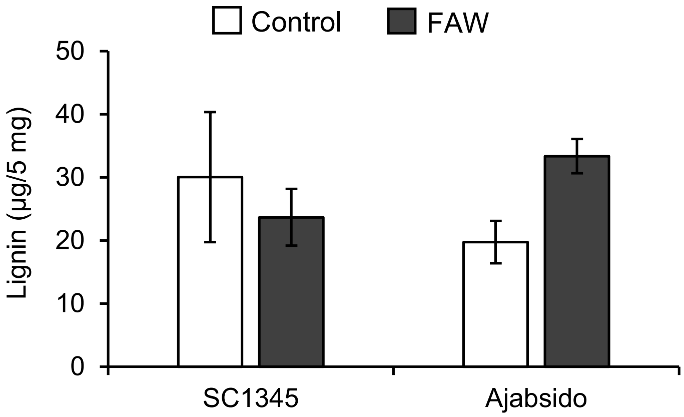

Supp. Fig. S6 Quantification of lignin content in leaves of SC1345 and Ajabsido plants after feeding by fall armyworm (FAW) larvae for 10 days. Plants that were not infested with FAW were used as controls (n = 3-4). Error bars represent ± SE. No significant differences were observed among any of the treatments ($P > 0.05$).

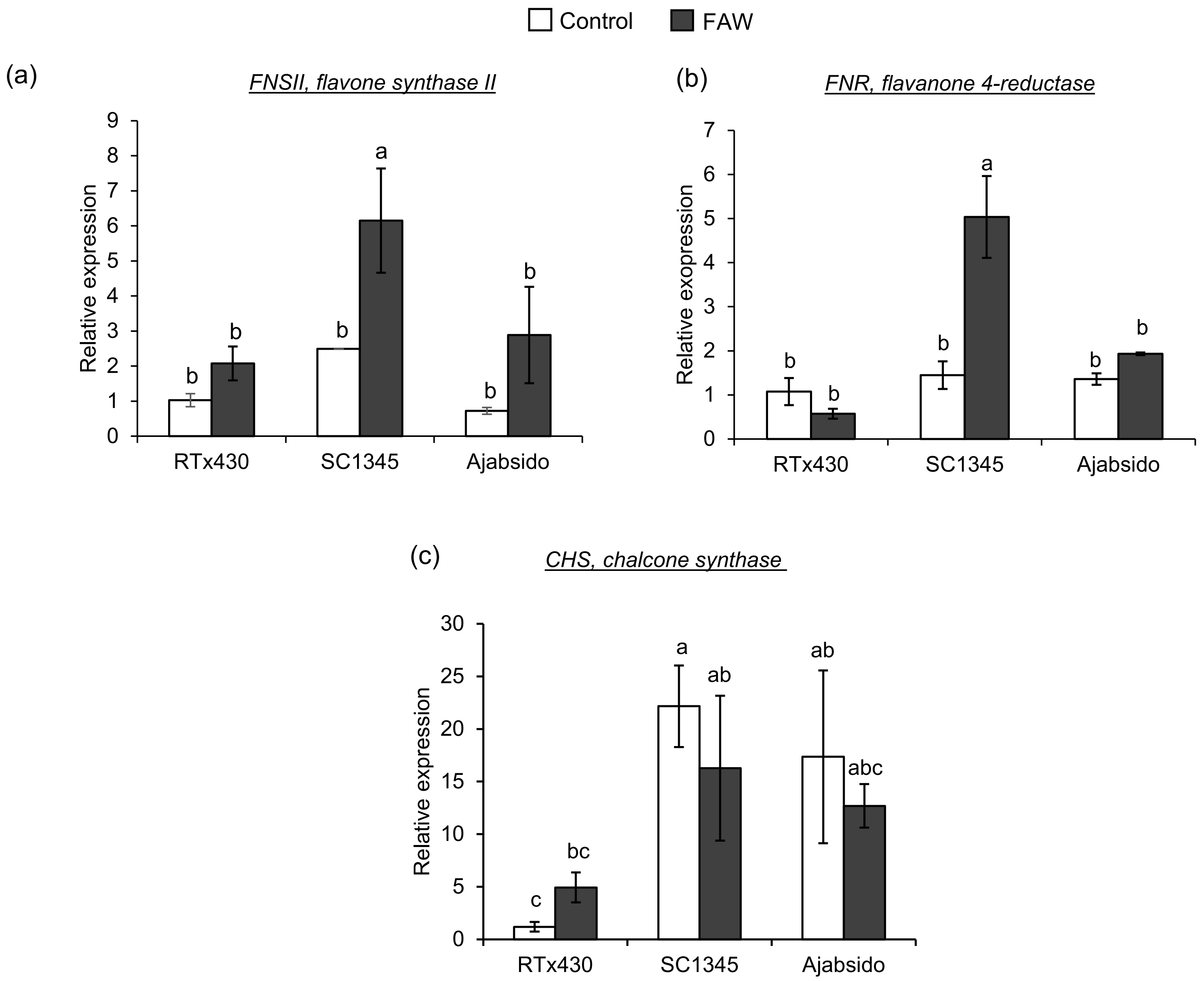

Supp. Fig. S7 Fall armyworm (FAW) feeding enhances the expression of genes involved in flavonoid biosynthetic pathway in sorghum. RT-qPCR analysis of flavonoid pathway genes, (a) *FNSII*, flavone synthase II; (b) *FNR*, flavanone 4-reductase; (c) *CHS*, chalcone synthase, expression in leaves of sorghum RTx430, Ajabsido, and SC1345 plants after feeding by FAW larvae for 10 days. Plants that were not infested with FAW were used as controls (n = 3-4). Error bars represent ± SE. Different letters indicate significant difference relative to each other ($P < 0.05$).

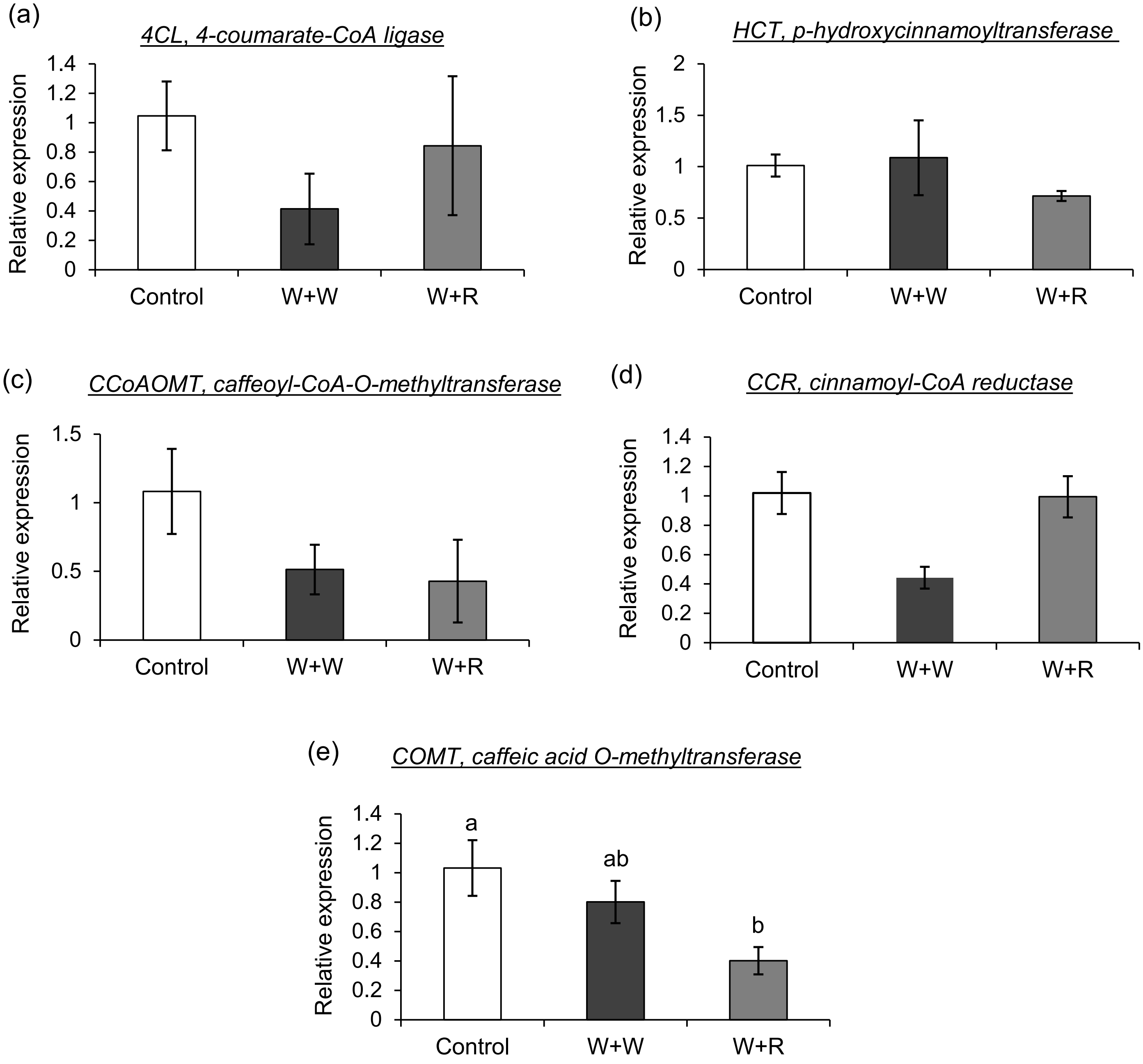

Supp. Fig. S8 Modulation of sorghum genes involved in monolignol biosynthetic pathway after application of fall armyworm (FAW) regurgitant on SC1345 plants. RT-qPCR analysis of lignin pathway genes in sorghum SC1345 plants after four applications of FAW regurgitant every 24 hours. (a) *4CL*, 4-coumarate: CoA ligase; (b) *HCT*, p-hydroxycinnamoyltransferase; (c) *CCoAOMT*, caffeoyl-CoA-O-methyltransferase; (d) *CCR*, cinnamoyl-CoA reductase; and (e) *COMT*, caffeic acid O-methyl transferase. Undamaged and wounded plants were used as controls (n = 3-4). Error bars represent ± SE. Different letters indicate significant difference relative to each other ($P < 0.05$). W + W, treatments in which water was applied to wounds; W + R, treatments in which FAW regurgitant was applied to wounds.

Supp. Table S1

RT-qPCR primers used to analyze expression levels of genes from the monolignol and flavonoid biosynthesis pathway where F refers to Forward Primer Sequence (5’ to 3’) and R refers to Reverse Primer Sequence (3’ to 5’). The CHS primers were taken from (Shih et al., 2006), which were derived from the conserved region among the CHS gene members (Lo et al., 2002).

Gene Name	Gene ID	Orientation	Primer Sequence (5' to 3')
Phenylalanine ammonia lyase (PAL)	Sobic.004G220300	F	TCTACGGCGTCACCACGGGG
		R	ACCTCCGACGGCAGCGTGT
Cinnamate 4-hydroxylase (C4H)	Sobic.002G126600	F	ACCTCAACCGCTGCCATGACC
		R	GCACCGGATCTCACCAGTCTGAG
4-coumarate: CoA ligase (4CL)	Sobic.004G062500	F	CCGAAGGCTCTGAAGTCACCGAG
		R	AGGATCTTGCCGGACGGGTTC
Cinnamoyl-CoA reductase (CCR)	Sobic.007G141200	F	GGGAACGGGCAGACCGTGTG
		R	GGTGCGCGTTCTTCGGGTCA
Hydroxycinnamoyl transferase (HCT)	Sobic.004G212300	F	CTTCTCCGACGACACCGCCTTC
		R	TAGCCACGCCACCGCATTTGAA
Ferulate 5-hydroxylase (F5H)	Sobic.001G196300	F	CAGAACACGCTCCGCCTCAC
		R	TCTCCGTCCCGCCAAACATCA
Caffeoyl-CoA O-methyltransferase (CCoAOMT)	Sobic.010G052200	F	AGATCACCGCCAAGCACCCA
		R	GCGCCGATGAGCTTGATGAGC
Caffeic O-methyltransferase (COMT)	Sobic.007G047300	F	GCTCACCCCTAACGAGGACGG
		R	GCACCGCGTCCTTCAGGTAGTA
Cinnamic alcohol dehydrogenase (CAD)	Sobic.004G071000	F	GTGGTGAAGGTGCTCTACTG
		R	CGTTGTAGGACCAGATCTTC
α-tubulin gene (α-Tub)	Sobic.001G107200	F	TCGGAAACGCGTGCTGGGAG
		R	AGCATCGTCACCTCCCCCAA
Dihydroflavonol-4-reductase (DFR3)	Sobic.004G050200	F	CGCAAGACCACCGTCTTCTT
		R	GGTAGCTTTTCCTGTTGCCG

Flavone synthase II (FNSII)	Sobic.002G000400	F	CGCAAGACCACCGTCTTCTT
		R	GGTAGCTTTTCCTGTTGCCG
flavanone 4-reductase (FNR)	Sobic.006G226800	F	GGGTAACAAGAAGACGATGAAGA
		R	CTGGATCCTGTGCCTCGAAGT
Chalcone Synthase (CHS)	-------	F	AGGTGGAGGCCAAGGTC
		R	CGTCGAGGATGAAGAGCAC
